# Control of competence by related non-coding csRNAs in *Streptococcus pneumoniae* R6

**DOI:** 10.3389/fgene.2015.00246

**Published:** 2015-07-20

**Authors:** Anke Laux, Anne Sexauer, Dineshan Sivaselvarajah, Anne Kaysen, Reinhold Brückner

**Affiliations:** Department of Microbiology, University of KaiserslauternKaiserslautern, Germany

**Keywords:** *Streptococcus pneumoniae*, two-component regulatory system CiaRH, small non-coding csRNAs, post-transcriptional regulation, competence development

## Abstract

The two-component regulatory system CiaRH of *Streptococcus pneumoniae* is involved in β-lactam resistance, maintenance of cell integrity, bacteriocin production, host colonization, virulence, and competence. The response regulator CiaR controls, among other genes, expression of five highly similar small non-coding RNAs, designated csRNAs. These csRNAs control competence development by targeting *comC*, encoding the precursor of the competence stimulating peptide, which is essential to initiate the regulatory cascade leading to competence. In addition, another gene product of the CiaR regulon, the serine protease HtrA, is also involved in competence control. In the absence of HtrA, five csRNAs could suppress competence, but one csRNA alone was not effective. To determine if all csRNAs are needed, reporter gene fusions to competence genes were used to monitor competence gene expression in the presence of different csRNAs. These experiments showed that two csRNAs were not enough to prevent competence, but combinations of three csRNAs, csRNA1,2,3, or csRNA1,2,4 were sufficient. In *S*. *pneumoniae* strains expressing only csRNA5, a surprising positive effect was detected on the level of early competence gene expression. Hence, the role of the csRNAs in competence regulation is more complex than anticipated. Mutations in *comC* (*comC8*) partially disrupting predicted complementarity to the csRNAs led to competence even in the presence of all csRNAs. Reconstitution of csRNA complementarity to *comC8* restored competence suppression. Again, more than one csRNA was needed. In this case, even two mutated csRNAs complementary to *comC8*, csRNA1–8 and csRNA2–8, were suppressive. In conclusion, competence in *S*. *pneumoniae* is additively controlled by the csRNAs via post-transcriptional regulation of *comC*.

## Introduction

The human pathogen *Streptococcus pneumoniae* shows a remarkable genomic plasticity (Croucher et al., 2012, 2014) and is a paradigm for bacteria that are able to undergo natural genetic transformation (for recent reviews see Johnston et al., 2014; Straume et al., 2014). The ability of *S*. *pneumoniae* to develop genetic competence is a highly regulated process depending on a variety of internal and external signals. Of these signals, the secreted peptide pheromone CSP (competence stimulating peptide) is absolutely essential. CSP is the processed extracellular form of the *comC* gene product encoded as the first gene of the *comCDE* operon (Håvarstein et al., 1995; Pestova et al., 1996). Pre-CSP is exported and processed by a dedicated ABC transporter ComAB (Hui and Morrison, 1991; Hui et al., 1995). CSP activates the two-component regulatory system ComDE by binding to the membrane-spanning histidine kinase ComD (Håvarstein et al., 1996). At the end of this initiating regulatory cascade, the response regulator ComE is phosphorylated (Martin et al., 2013) and helps to transcribe early competence genes, among them *comAB*, *comCDE*, and *comX1/comX2* (Peterson et al., 2004). Enhanced transcription of *comAB* and *comCDE* results in an autocatalytic loop ensuring competence development throughout the population. The identical genes *comX1*/*comX2* in turn encode the alternative sigma factor ComX, which directs transcription of late competence genes (Lee and Morrison, 1999). Proteins encoded by this class of genes are required for uptake of DNA and subsequent recombination (Claverys et al., 2009; Laurenceau et al., 2013).

The early regulatory steps constitute an extremely sensitive switch to the competence state, which is defined by the threshold to turn on the initiating autocatalytic loop. Processes interfering with CSP accumulation or CSP binding to the ComD sensor will inevitably modulate competence development. Consequently, quite a number of physiological parameters affect competence (Claverys and Håvarstein, 2002). Remarkably, stress conditions such as treatment with antibiotics may induce competence by various mechanisms (Prudhomme et al., 2006; Stevens et al., 2011; Gagne et al., 2013; Slager et al., 2014).

Besides the essential regulatory devices summarized above, competence development is modulated by other regulators such as the two-component regulatory systems CiaRH, WalRK, and the serine/threonine kinase StkP (Guenzi et al., 1994; Echenique and Trombe, 2001; Echenique et al., 2004; Saskova et al., 2007). While the regulatory link of WalRK or StkP to competence has not been established, several gene products of the CiaR regulon have been shown to interfere with CSP accumulation (Sebert et al., 2005; Cassone et al., 2012; Schnorpfeil et al., 2013). The cell surface-associated serine protease HtrA is apparently able to cleave CSP under certain conditions (Stevens et al., 2011) and five small non-coding csRNAs (cia-dependent small RNAs) regulate *comC* post-transcriptionally thereby reducing CSP production (Schnorpfeil et al., 2013). The csRNAs range between 87 and 151 nt in size and show a high degree of sequence similarity to each other (Halfmann et al., 2007b). Sequences potentially base-pairing to ribosome binding sites and AUG start codons are present in all csRNAs and are believed to be important for their regulatory function (Schnorpfeil et al., 2013). The csRNAs belong to an expanding class of small RNAs (sRNAs), which were designated sibling sRNAs in a recent review article (Caswell et al., 2014). Six targets for the csRNAs have been identified so far (Schnorpfeil et al., 2013). Besides *comC*, four genes encoding transporters and one gene coding for a DNA-binding protein are regulated. For three tested targets, among them *comC*, it was determined that the csRNAs act additively. Each csRNA could downregulate the respective genes, but each single csRNA was not as effective as all five csRNAs (Schnorpfeil et al., 2013). Combinations of csRNAs had not been tested in these experiments.

In this communication we determined the influence of csRNA combinations on competence gene expression. The results of these experiments showed that all five csRNAs are not needed for competence suppression. Instead, several combinations of three csRNAs were sufficient. In addition, a special role in early competence gene regulation was detected for csRNA5.

## Materials and Methods

### Bacterial Strains and Growth Conditions

The *S*. *pneumoniae* strains in this work are derived from *S. pneumoniae* R6 (Ottolenghi and Hotchkiss, 1962) and are listed in **Table 1**. Strains expressing single csRNAs or combinations were obtained by three approaches. First, a series of marker-less Cheshire deletions (Weng et al., 2009) were introduced as described previously (Schnorpfeil et al., 2013). Secondly, since for *ccnAB*, the genes encoding csRNA1 and csRNA2, only one Cheshire construct was available deleting both *csRNA* genes, *ermAM* was applied for individual *ccnA* or *ccnB* inactivation with constructions that were available from previous work (Halfmann et al., 2007b). And finally, to avoid time consuming repeated Cheshire constructions, some strains were obtained by ectopic expression of csRNA genes carried on integrative plasmids (Schnorpfeil et al., 2013). This approach results in csRNA expression levels that are indistinguishable from the wild type situation (Schnorpfeil et al., 2013). The *comC8* mutant allele uncoupling competence development from csRNA control (Schnorpfeil et al., 2013) was introduced into a csRNA- and HtrA-deficient strain (RKL558, **Table 1**), which had been transformed with *rpsl41* DNA to yield a streptomycin resistant derivative (RKL362). The subsequent Janus procedure (Sung et al., 2001) for *comC8* replacement was carried out as described (Schnorpfeil et al., 2013). First, the *comC*::*janus* fragment was integrated into RKL362 to yield RKL745, which is streptomycin-sensitive and kanamycin-resistant. The final *comC8* construct was introduced to RKL745 resulting in the streptomycin-resistant, kanamycin-sensitive RKL746 (**Table 1**). Complementing csRNA variants were then introduced into RKL746 (see below).

**Table 1:** *Streptococcus pneumoniae* strains used in this study.

Strain^a^	Characteristics	Reference
RKL229	*ccnAB::lox72, ccnC::lox72, ccnD::lox72, ccnE::lox72 (ΔccnA-E)*	Schnorpfeil et al. (2013)
RKL557	*htrA::aphlll*	Schnorpfeil et al. (2013)
RKL558	*ccnAB::lox72, ccnC::lox72, ccnD::lox72, ccnE::lox72 (ΔccnA-E), htrA::aphlll*	Schnorpfeil et al. (2013)
RKL362	*(ΔccnA-E), rpsL41*	This work
RKL688	*ccnB::ermAM, ccnC::lox72, ccnD::lox72, ccnE::lox72, htrA::aphlll*	This work
RKL689	*ccnA::ermAM, ccnC::lox72, ccnD::lox72, ccnE::lox72, htrA::aphlll*	This work
RKL690	*ccnAB::lox72, ccnD::lox72, ccnE::lox72, htrA::aphlll*	This work
RKL691	*ccnAB::lox72, ccnC::lox72, ccnE::lox72, htrA::aphlll*	This work
RKL692	*ccnAB::lox72, ccnC::lox72, ccnD::lox72, htrA::aphlll*	This work
RKL693	*ccnAB::lox72, ccnC::lox72, htrA::aphlll*	This work
RKL694	*ccnC::lox72, ccnD::lox72, ccnE::lox72, htrA::aphlll*	This work
RKL695	*ccnD:: lox72, ccnE:: lox72, htrA:: aphlll*	This work
RKL745	*ΔccnA-E, rpsL41, comC: janus*	This work
RKL746	*ΔccnA-E, rpsL41, comC8*	This work
RKL913	*ccnC::lox72, ccnD::lox72, ccnE::lox72, htrA::aphlll bgaA tmp-ccnD-spr0568*	This work
RKL914	ccnB::ermAM ccnC::lox7*2, ccnD::lox72, ccnE::lox72, htrA::aphlll, bgaA tmp-ccnC-spr0568*	This work
RKL915	ccnB::ermAM ccnC::lox7*2, ccnD::lox72, ccnE::lox72, htrA::aphlll, bgaA tmp-ccnD-spr0568*	This work
RKL916	*ccnA::ermAM, ccnC::lox72, ccnD::lox72, ccnE::lox72, htrA::aphlll, bgaA tmp-ccnC-spr0568*	This work
RKL917	*ccnA::ermAM, ccnC::lox72, ccnD::lox72, ccnE::lox72, htrA::aphlll, bgaA tmp-ccnD-spr0568*	This work
RKL918	*ccnAB*:: *lox72, ccnD*:: *lox72, ccnE:*: *lox72, htrA*:: *aphlll, bgaA tmp-ccnD-spr0568*	This work
RKL939	*ΔccnA-E, rpsL41, comC8, htrA::aphlll*	This work
RKL941	*ΔccnA-E, rpsL41, comC8, htrA::aphlll, bgaA tmp-ccnA-8-spr0568*	This work
RKL942	*ΔccnA-E, rpsL41, comC8, htrA::aphlll, bgaA tmp-ccnAB-8-spr0568*	This work
RKL943	*ΔccnA-E, rpsL41, comC8, htrA::aphlll, bgaA tmp-ccnB-8-spr0568*	This work
RKL967	*ccnBr.ermAM, ccnC::lox72, ccnD::lox72; ccnE::lox72 htrA::aphlll, bgaA tmp-ccnE-spr0568*	This work
RKL968	*ccnAr.ermAM, ccnC::lox72, ccnD::lox72; ccnE::lox72 htrA::aphlll, bgaA tmp-ccnE-spr0568*	This work
RKL969	*ccnC:: lox72, ccnD:: lox72; ccnE:: lox72 htrA::aphlll, bgaA tmp-ccnE-spr0568*	This work

*^a^All strains are R6 derivatives.*

For strain preservation or transformation, *S. pneumoniae* was grown at 37°C in C + Y medium (Lacks and Hotchkiss, 1960; Schnorpfeil et al., 2013). The competence expression tests were performed in brain–heart infusion (BHI, pH 7.4) using BHI, pH 7.0 for inoculation. BHI was purchased from Becton Dickinson, France. Growth of *S. pneumoniae* was monitored by measuring optical density of 600 nm (OD_600_).

Plasmid cloning was performed in *Escherichia coli* DH5α [ϕ80d*lacZ*ΔM15 Δ(*lacZYA*-*argF*) *recA1 endA1 hsdR17 supE44 thi-1 gyrA96 phoA relA1*]. *E*. *coli* strains were grown in LB medium.

### Construction of Promoter Probe Plasmids and Transformation

To monitor competence development, two promoter fusions were constructed. One promoter originated from the genes *comX1/2* and represents the early class of competence-specific promoters dependent on ComE (Peterson et al., 2004). Since *comX1/2* are identical genes including identical promoter regions the resulting promoter is designated P*_comX_*. This promoter was obtained by PCR using primers ComX1Eco (CTCGAATTCATGCACTATCCTATGAAGTAAAGTC) and comX-Prom-bam (CAGGGATCCTTTCTAGATACAGTCTAACAGATTAGAAAACAC). The resulting fragment was cloned into promoter probe plasmid pPP2 (Halfmann et al., 2007a) to yield plasmid pPPcomX. The other promoter is from the *cibABC* operon and constitutes a late competence promoter dependent on the alternative sigma factor ComX (Peterson et al., 2004). To clone the *cibA* promoter, the primers CibA-1 (GGCGCATGCAGACAAGAGTGCCCTCACTTAAC) and CibAprom (GGCGGATCCCTTGTTCACTTTTATATTCGGAAAAG) were used to yield pPP2cibA.

Plasmid pPC2 carrying another early competence promoter (P*_comC_*) was described previously (Halfmann et al., 2007a). Upon integration of the promoter fusion plasmids, the genotype of the resulting strains was as follows: *bgaA*::*tetM*-P*_comX_*-*lacZ*, *bgaA*::*tetM*-P*_cibA_*-*lacZ, bgaA*::*tetM*-P*_comC_*-*lacZ.*

To introduce the plasmids into *S*. *pneumoniae*, cells were grown in C + Y medium and were transformed as described (Schnorpfeil et al., 2013). *S. pneumoniae* transformants with pPP derivatives were selected on plates containing D-blood agar (Alloing et al., 1996) at 37°C with 2 μg tetracycline per ml. Transformation of *E*. *coli* was carried out according to Hanahan (1983).

### Expression of *ccn* Genes Encoding csRNAs Complementary to *comC8*

The *comC8* gene variant relieved competence regulation from csRNA-mediated control (Schnorpfeil et al., 2013). Mutations were introduced into csRNA1 and csRNA2, that restored complementarity to *comC8* (**Figure 3**). The mutated csRNA genes, csRNA1–8 and csRNA2–8, were obtained after gene synthesis cloned into pUC57 (Genewiz, South Plainfield, NJ, USA). Two plasmids contained single mutated csRNA genes, the third contained csRNA1–8 and csRNA2–8 in tandem as found for the wild type *ccnAB* genes in the *S. pneumoniae* genome (Halfmann et al., 2007b). The mutated csRNA genes were moved alone or in combination to the integrative plasmid pSW1 (Denapaite and Hakenbeck, 2011) to yield plasmids pCcnA-8, pCcnB-8, and pCcnA-8-ccnB-8. Plasmids were introduced to the csRNA- and HtrA-deficient strain carrying the *comC8* allele RKL746 by selection with 100 μg/ml trimethoprim. Correct integration was determined by PCR and sequencing.

### Determination of β-Galactosidase Activity

To follow the onset of competence gene expression, strains harboring promoter-β-galactosidase gene (*lacZ*) fusions in the genome (Halfmann et al., 2007b) were assayed for β-galactosidase activity during growth as described (Weng et al., 2013). To estimate the significance of the different time points of β-galactosidase induction, Student’s *t*-tests were performed. Differences were considered significant, when *p* < 0.05 was given.

## Results

### Competence Gene Expression in the Presence of Single csRNAs

**FIGURE 1:**
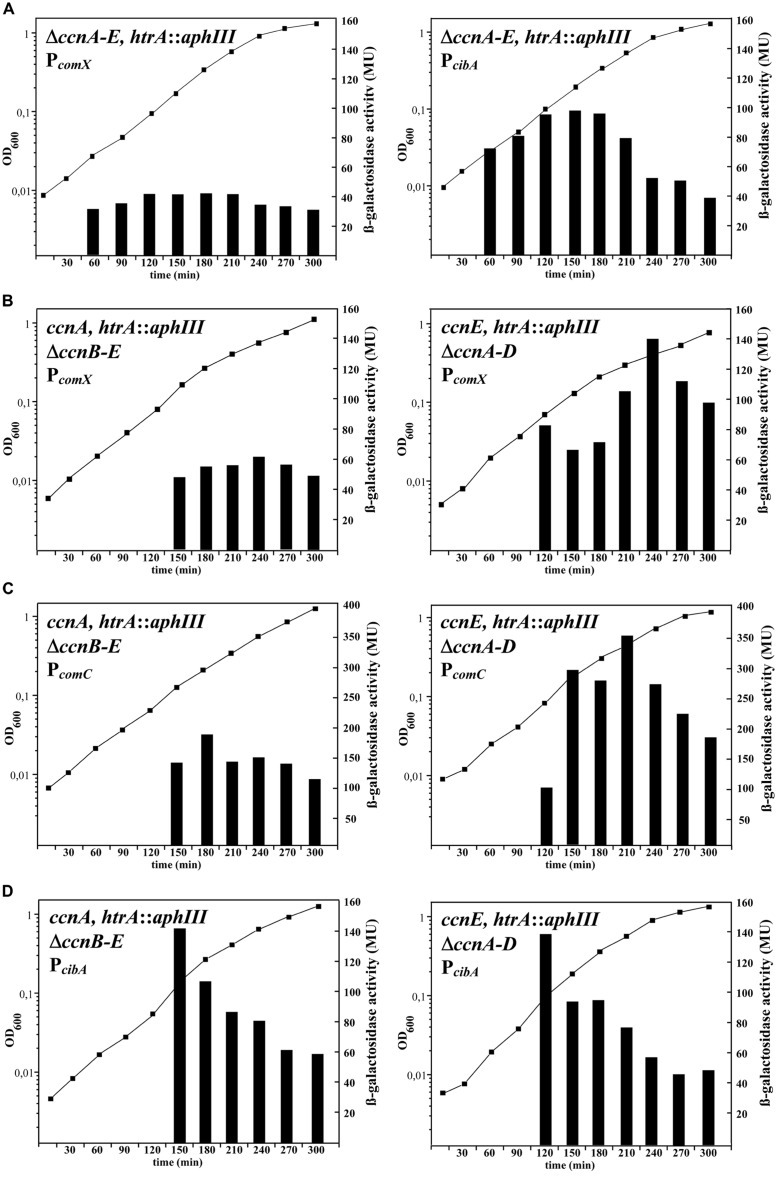
**Time course of competence gene expression in *Streptococcus pneumoniae* strains expressing different csRNAs.** The strains were grown in brain heart infusion medium and β-galactosidase activities were determined in 30 min. intervals. β-galactosidase activities of representative experiments are shown along with the growth curve determined by measuring OD_600_. All strains contained a genomic integration of a competence promoter fused to the promoterless β-galactosidase gene *lacZ* of *Escherichia coli* and a disrupted protease gene *htrA* (*htrA::aphIII*). Relevant genetic characteristics are indicated. **(A)** Activity of early and late competence promoters in strains without csRNAs. The early competence gene promoter of *comX* and the late competence gene promoter of *cibA* were fused to *lacZ* and integrated into RKL 558 (*ccnA*:: *lox72, ccnB*:: *lox72, ccnC*::*lox72, ccnD*::*lox72, ccnE*::*lox72, htrA::aphIII).* The promoter fusion integrations were: *bgaA*::*tetM*-P*_comX_*-*lacZ* (left), *bgaA*::*tetM*-P*_cibA_*-*lacZ* (right). **(B)** Activity of the early *comX* promoter in strains with csRNA1 or csRNA5. The early competence gene promoter of *comX* was fused to *lacZ* and integrated into RKL688 (*ccnB*::*ermAM, ccnC*::*lox72, ccnD*::*lox72, ccnE*::*lox72, htrA::aphIII*, left) or RKL692 (*ccnAB*::*lox72*, *ccnC*::*lox72*, *ccnD*::*lox72*, *htrA*::*aphIII*, right). Both strains contained the integrated P*_comX_*-*lacZ* fusion: *bgaA*::*tetM*-P*_comX_*-*lacZ*. **(C)** Activity of the early *comC* promoter in strains with csRNA1 or csRNA5. The early competence gene promoter of *comC* was fused to *lacZ* and integrated into the same strains as in **(B)**. (RKL688; *ccnB*::*ermAM, ccnC*::*lox72, ccnD*::*lox72, ccnE*::*lox72, htrA::aphIII*, left, or RKL692; *ccnAB*::*lox72*, *ccnC*::*lox72*, *ccnD*::*lox72*, *htrA*::*aphIII*, right). Both strains contained the integrated P*_comC_*-*lacZ* fusion: *bgaA*::*tetM*-P*_comX_*-*lacZ*. **(D)** Activity of the late *cibA* promoter in strains with csRNA1 or csRNA5. The late competence gene promoter of *cibA* was fused to *lacZ* and integrated into the same strains as in **(B,C)**. (RKL688; *ccnB*::*ermAM, ccnC*::*lox72, ccnD*::*lox72, ccnE*::*lox72, htrA::aphIII*, left, or RKL692; *ccnAB*::*lox72*, *ccnC*::*lox72*, *ccnD*::*lox72*, *htrA*::*aphIII*, right). Both strains contained the integrated P*_cibA_*-*lacZ* fusion: *bgaA*::*tetM*-P*_comX_*-*lacZ*.

As detailed above, the two-component regulatory system CiaRH is one of the most important additional regulatory factors modulating competence development (Sebert et al., 2005; Schnorpfeil et al., 2013). Depending on the growth conditions and the expression state of CiaRH, the protease HtrA, and/or the five small csRNA are able to suppress competence. Transformation experiments performed in competence non-permissive BHI medium showed that inactivation of *htrA* and the csRNA genes *ccnA-D* is needed to allow competence to develop (Schnorpfeil et al., 2013). To monitor competence development more conveniently, promoter fusions to the *E*. *coli* β-galactosidase gene *lacZ* were constructed using promoters of *comX* and the *cibABC* operon as representatives for early (*comX*) and late (*cibA*) competence genes (Peterson et al., 2004). After integration of these promoter-*lacZ* fusions into the genome of *S*. *pneumoniae*, β-galactosidase activities were measured during growth in BHI medium. As predicted from the previous transformation experiments, *lacZ* expression driven by both promoters was only detectable in the absence of the csRNAs and the protease HtrA (**Figure 1A**). Expression of the competence genes started very early in growth at an OD_600_ around 0.03 (**Figure 1A**; **Table 2**). Due to the 30 min interval of measurements, the time difference between early and late gene expression of about 5–7 min (Peterson et al., 2004) is not detected (**Figure 1A**). In addition, competence shut-off is not clearly seen in these experiments, because of the rather stable β-galactosidase, which is still active after transcription of its gene ceases. Since CiaRH is involved in controlling early steps of competence gene expression, only their induction is relevant and, as shown in **Figure 1A**, clearly detected.

**Table 2:** Expression of competence genes in *S. pneumoniae* strains with different csRNA genes.

strain^a^	csRNA genes	csRNAs	*comX* expression	induction of *comX* expression at OD_600_^b^
RKL557	*ccnABCDE*	csRNAl,2,3,4,5	-^c^	na
RKL558	-	-	+	0.03 ± 0.02
RKL688	*ccnA*	csRNAl	+	0.15 ± 0.01
RKL689	*ccnB*	csRNA2	+	0.17 ± 0.002
RKL690	*ccnC*	csRNA3	+	0.21 ± 0.001
RKL691	*ccnD*	csRNA4	+	0.30 ± 0.001
RKL692	*ccnE*	csRNA5	+	0.09 ± 0.005
RKL694	*ccnAB*	csRNAl,2	+	0.36 ± 0.07
RKL914	*ccnAC*	csRNAl,3	+	0.36 ± 0.05
RKL915	*ccnAD*	csRNAl,4	+	0.31 ± 0.08
RKL967	*ccnAE*	csRNAl,5	+	0.18 ± 0.05
RKL916	*ccnBC*	csRNA2,3	+	0.30 ± 0.07
RKL917	*ccnBD*	csRNA2,4	+	0.36 ± 0.01
RKL968	*ccnBE*	csRNA2,5	+	0.19 ± 0.03
RKL918	*ccnCD*	csRNA3,4	+	0.31 ± 0.03
RKL693	*ccnDE*	csRNA4,5	+	0.25 ± 0.04
RKL695	*ccnABC*	csRNAl,2,3	-^c^	na
RKL913	*ccnABD*	csRNAl,2,4	-^c^	na
RKL969	*ccnABE*	csRNAl,2,5	+	0.38 ± 0.1

*^a^All strains are serine protease HtrA deficient (htrA::aphIII) and contain a P_comX_-lacZ fusion in the genome (bgaA::tetM-P_comX_-lacZ). They were grown in BHI medium.*

*^b^The onset of comX expression was defined as the time point when more than 10 Miller units (MU) β-galactosidase were reached. Uninduced P_comX_-meditaed expression was below 1 MU. Values of at least two independent cultures are shown along with SDs. Timing of β-galactosidase expression was significantly different (p < 0.05) in all strains compared to RKL558.*

*^c^Expression around or below β-galactosidase activity detection level (∼1 MU). Na, not applicable.*

**FIGURE 2:**
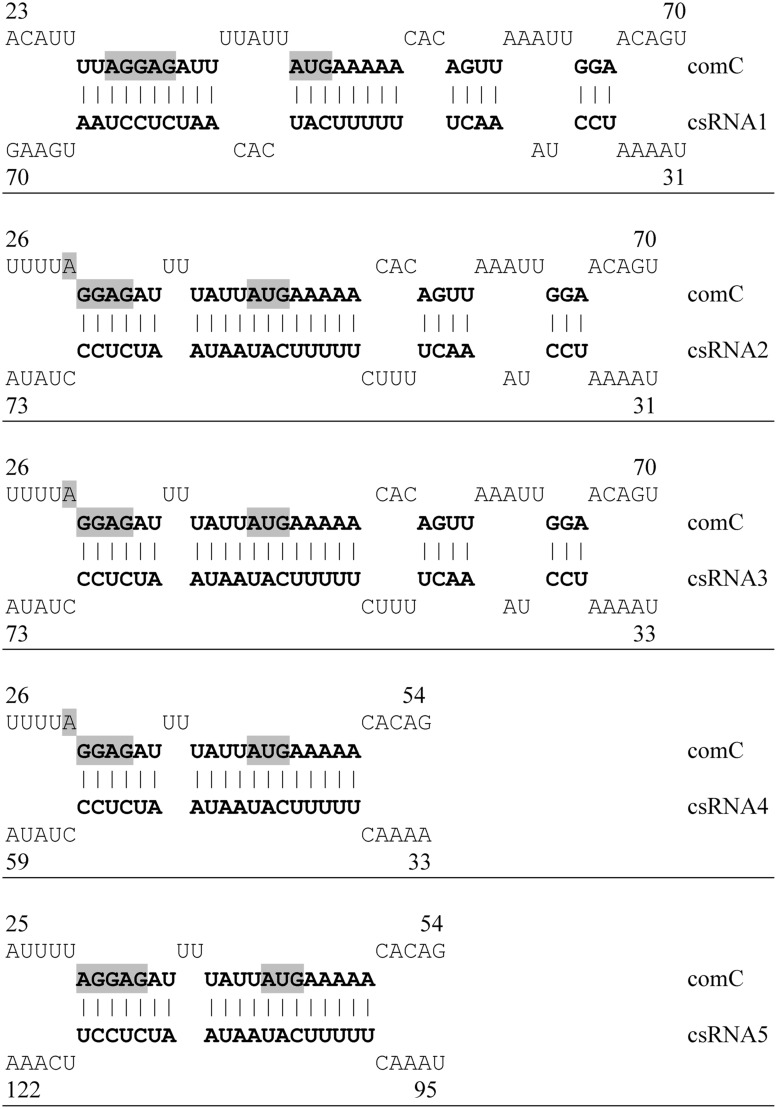
**Predicted interactions of csRNAs with *comC* mRNA.** Base-pairing of the csRNAs with *comC* mRNA according to IntaRNA predictions are shown (Busch et al., 2008). Predicted base pairs are shown in bold face, the Shine Dalgarno (SD) sequence and the start codon of *comC* are shaded in gray. The numbers refer to the bases counted from the transcriptional start sites of the *csRNAs* and *comC*. Putative interactions of all csRNAs with *comC* mRNA are shown.

To determine which and how many csRNAs are needed to block competence, strains were constructed expressing single csRNAs and various combinations (**Table 1**). Each of the csRNAs is predicted to bind to *comC* (**Figure 2**) and could reduce expression from a *comC*′–′*lacZ* translational fusion (Schnorpfeil et al., 2013). Competence gene expression was measured during growth in BHI medium as shown for the P*_comX_*-*lacZ* fusion and strains expressing csRNA1 or csRNA5 as examples in **Figure 1B**. Individual csRNAs were not able to suppress competence gene expression, which is in accordance with previous transformation tests (Schnorpfeil et al., 2013). However, the cell density, at which induction occurred, varied, and was substantially higher than in the strain without csRNAs (**Figures 1A,B**; **Table 2**). With csRNA4, expression started at OD_600_ of 0.3, with csRNA5 already at 0.09, and with the other csRNAs in between these cell densities (**Table 2**). Thus, although single csRNAs are not able to turn down competence, they are able to delay its start.

### Competence Gene Expression in the Presence of Multiple csRNAs

Next we asked how subsets of csRNAs could affect competence in *S*. *pneumoniae.* To start with that analysis, strains were constructed expressing combinations of two csRNAs (**Table 1**). As shown in **Table 2**, none of the combinations was able to stop competence gene expression, not even the two csRNAs, csRNA3 and csRNA4, which alone could maximally delay competence induction. With most of the combinations, competence gene expression started at higher cell densities than with single csRNAs, moving competence to the mid-exponential growth phase.

Combining three csRNAs, for example csRNA1,2,3 or csRNA1,2,4 blocked competence completely. Therefore, not all csRNAs are needed and more than one combination can be effective. Combination of csRNA1,2, and 5 did not stop P_comX_ promoter activity, corroborating the weak negative effect of csRNA5 observed in cells with csRNA5 alone.

### A Special Role for csRNA5 on the Level of Early Competence Gene Expression

During the experiments detailed above, a remarkable effect on *comX* promoter activity was detected when csRNA5 was present (**Figure 1B**). The strength of P*_comX_*-mediated β-galactosidase expression was about twofold enhanced in the presence of csRNA5 compared to a strain with csRNA1. Strains expressing csRNA2 or csRNA3 showed the same P*_comX_*-mediated β-galactosidase expression level as the csRNA1 containing strain (data not shown). Only expression timing was different (**Table 2**). In *S*. *pneumoniae* with csRNA4, P*_comX_* promoter activity mediated β-galactosidase was slightly higher (1,4-fold). Therefore, besides acting negatively on the timing of competence, csRNA5 and to a lesser extent csRNA4 play a positive role in P*_comX_* expression. Enhanced P_comX_ promoter activity was also detected when csRNA5 was expressed with other csRNAs, e.g., with csRNA2,4, or 1,2, however, not with csRNA1 alone.

To determine if the csRNA5 effect is also detected with other early competence promoters, a *comC* promoter fusion was subjected to the same analysis as the *comX* promoter. As shown in **Figure 1C**, P*_comC_* activity was affected by the presence of csRNA1 or csRNA5 in the same way as P*_comX_*. It appears therefore, that the strength of early competence promoters are positively affected by the presence of csRNA5. Therefore, the role of csRNA5 on early competence gene expression is both, negative and positive.

A different picture emerged, when late competence gene expression was tested in strains with csRNA1 and csRNA5. Measuring *cibA* promoter activity as an example for a late competence gene did not reveal a significant difference in the presence of csRNA1 or csRNA5 (**Figure 1D**). The observed positive effect of csRNA5 on the level of early competence gene expression is not passed on to late competence gene expression, most likely by elaborate feed-back control implemented in competence development (Johnston et al., 2014).

### Complementation of a csRNA-Insensitive *comC* Variant by Mutated csRNAs

**FIGURE 3:**
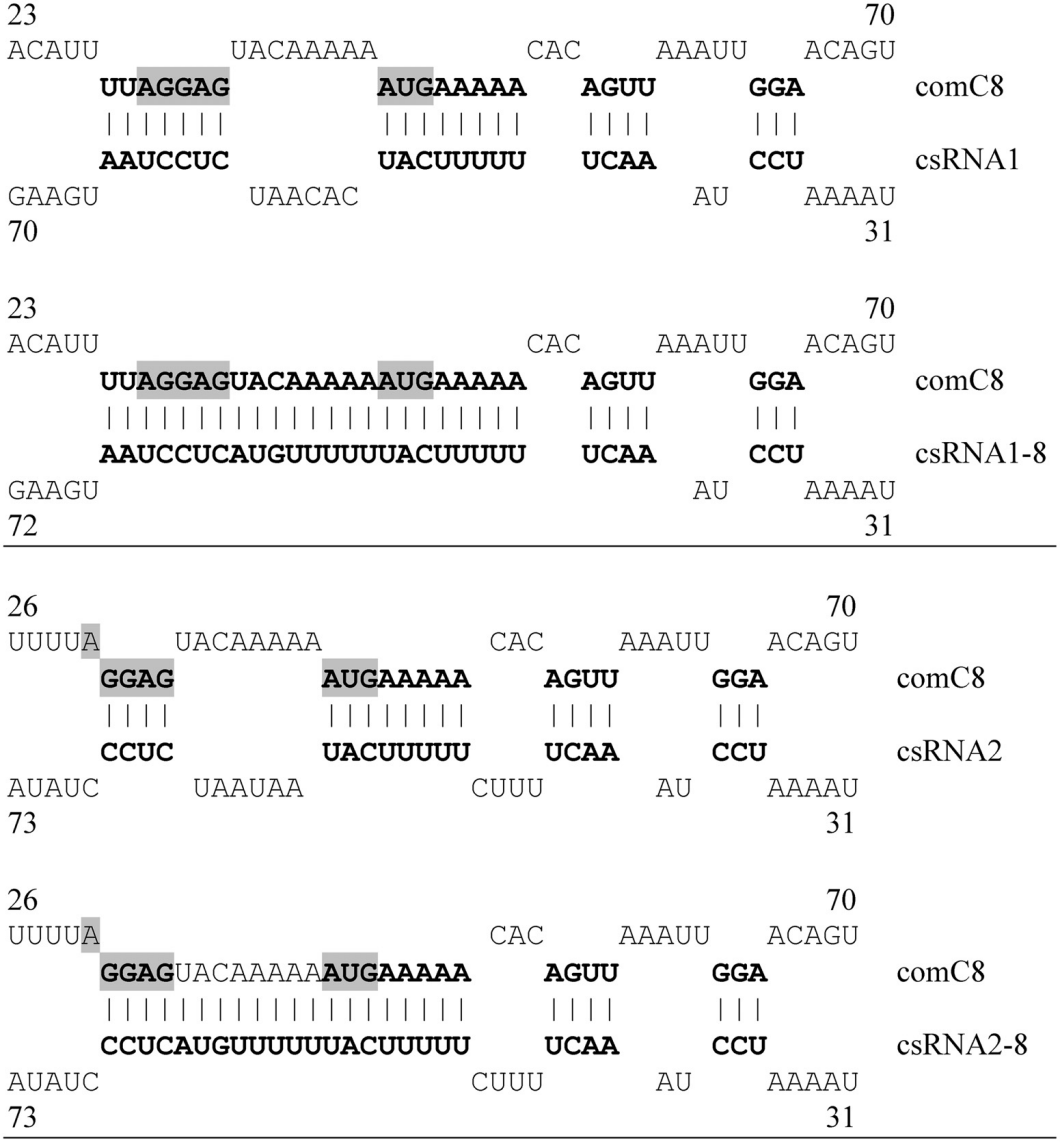
**Predicted interactions of *comC* mRNA with wild type csRNA1, csRNA2, and mutant csRNA1–8, csRNA2–8.** Base-pairing of the csRNAs with *comC8* mRNA according to IntaRNA predictions are shown (Busch et al., 2008). Base pairs are shown in bold face, the SD sequence and the start codon of *comC* are shaded. Complementarity of *comC8* to csRNA1 and csRNA2 is abolished between the SD sequence and AUG. Mutations in csRNA1–8 and csRNA2–8 restored complementarity. The numbers refer to the bases counted from the transcriptional start sites of the *csRNAs* and *comC8*.

In our previous work, a mutant *comC* gene, *comC8*, was constructed, which showed greatly diminished post-transcriptional control by the csRNAs (Schnorpfeil et al., 2013). Replacing *comC* by *comC8* resulted in strains that were able to develop competence even in the presence of all csRNAs (Schnorpfeil et al., 2013). Complementarity of *comC8* to the csRNAs had been reduced by mutations between the Shine-Dalgarno sequence (Shine and Dalgarno, 1974) and the start codon AUG (**Figure 3**). If regulation of competence by the csRNAs is indeed a direct consequence of csRNA–*comC* interaction, mutations in csRNAs restoring complementarity to *comC8* should re-establish csRNA-mediated competence control.

Therefore, mutated csRNA genes were constructed with changes complementary to *comC8* (**Figure 3**). As a start, csRNA1 and csRNA2 were mutated, since it could not be expected that a single modified csRNA could block competence alone. The mutated csRNAs, csRNA1–8 and csRNA2–8, were cloned alone and in combination to an integrative plasmid, which was used for ectopic expression of these csRNAs in a csRNA-deficient background (**Table 1**). To monitor competence development the late *cibA* promoter fusion was applied. With single mutated csRNAs, at least a delay in competence gene expression was expected and combinations could perhaps block competence.

As shown in **Table 3**, csRNA1–8 as well as csRNA2–8 could clearly delay competence gene expression. While expression started at OD_600_ of 0.09 without any csRNA, competence gene expression occurred at OD_600_ of 0.38 and 0.32 with csRNA1–8 or csRNA2–8, respectively. Thus, both mutated csRNAs similarly delayed competence, when expressed alone. In combination, they could even prevent competence development (**Table 3**), which is in contrast to the wild type, where three csRNAs were needed. Enhanced downregulation by csRNA1–8 and csRNA2–8 may be due to the enlarged region of complementarity introduced into the mutated csRNAs (**Figure 3**). The results of these experiments are completely consistent with the previous identification of the csRNAs as mediators of *comC* control and as one link of the CiaRH system to competence regulation (Schnorpfeil et al., 2013).

**Table 3:** Expression of competence genes in *S. pneumoniae comC8* strains with mutated csRNA genes.

strain^a^	csRNA genes	csRNAs	*cibA* expression	Induction of *cibA* expression at OD_600_^b^
RKL939	-	-	+	0.09 ± 0.04
RKL941	*ccnA-8*	csRNAl–	+	0.38 ± 0.08
RKL943	*ccnB-8*	csRNA2–	+	0.32 ± 0.1
RKL942	*ccnA-8, ccnB-8*	csRNAl–8,2–8	-^c^	na

*^a^All strains are serine protease HtrA deficient (htrA::aphIII), have the comC8 allele, which is no longer subject to csRNA control, and contain a P_cibA_-lacZ fusion in the genome (bgaA::tetM-P_cibA_-lacZ). They were grown in BHI medium.*

*^b^The onset of cibA expression was defined as the time point when more than 10 MUs β-galactosidase were reached. Uninduced P_cibA_-meditaed expression was below 1 MU. Values of at least two independent cultures are shown along with standard deviations. Timing of β-galactosidase expression in strains RKL 941 and 943 was significantly different (p < 0.05) compared to RKL939. The difference between RKL941 and RKL943 was not significant.*

*^c^Expression around or below β-galactosidase activity detection level (∼1 MU). na, not applicable.*

## Discussion

The two-component regulatory system CiaRH acts negatively on competence by controlling CSP accumulation by two mechanisms. One relies on the serine protease HtrA, which is able to cleave secreted CSP (Stevens et al., 2011), and the second on five csRNAs to reduce CSP production. Expression of the protease gene *htrA* as well as the csRNA genes are absolutely dependent on CiaR (Halfmann et al., 2007b). Which of the two mechanisms is more important strongly depends on the growth conditions. In BHI medium, HtrA or the csRNAs are able to suppress transformability (Schnorpfeil et al., 2013). In cells grown in C + Y medium, however, none of these components is able to stop competence development. Upon overexpression of the csRNAs and HtrA due to a hyperactive CiaRH system (Müller et al., 2011), competence is abolished even in C + Y medium (Schnorpfeil et al., 2013). Under these conditions, only the csRNAs are able to stop competence development. The level of csRNA production is clearly one parameter defining the regulatory outcome, but is not always sufficient to explain csRNA effects. In BHI and C + Y media, the CiaRH system is similarly active (Halfmann et al., 2011), but competence is only blocked in BHI medium (Schnorpfeil et al., 2013). These differences may be explained assuming that the CSP threshold to initiate competence could vary under these conditions. How this threshold level is set and influenced by growth conditions, remains one of the unsolved mysteries of competence regulation in *S*. *pneumoniae*.

The results of the *comC8* complementation experiments clearly identify *comC* regulation as the cause for csRNA-mediated competence suppression. They also point to the region where base pairing between csRNAs and *comC* occurs (**Figures 2** and **3**). The csRNAs bind around the *comC* translation initiation region, which is fully consistent with their negative effect on CSP production. Predicted csRNA-*comC* interaction is identical for csRNA2 and csRNA3 (**Figure 2**), almost identical except for one A–U pair for csRNA4 and csRNA5, and unique for csRNA1 (**Figure 2**). Especially for csRNA4 and csRNA5, predicted *comC* binding and their effect on competence regulation are not in accordance. If the delay in cell density when competence gene expression starts in strains expressing only one csRNA is taken as a measure of their effectiveness of *comC* downregulation (**Table 2**), csRNA4 and csRNA5 act at opposite ends: csRNA4 is the most (OD_600_ 0.3) and csRNA5 the least (OD_600_ 0.09) efficient, although predicted binding to *comC* is slightly better for csRNA5 (**Figure 3**). Likewise, csRNA4 could best reduce expression from a *comC*′–′*lacZ* translational fusion, while csRNA5 was least effective (Schnorpfeil et al., 2013). Interestingly, ranking of the effects of the csRNAs on *com*C′–′*lacZ* or the timing of competence gene expression produced the same order: csRNA4 acted best, followed by csRNA1, csRNA2, csRNA3, and at the end csRNA5 (Schnorpfeil et al., 2013), (**Table 2**). Thus, *comC* regulation by the csRNAs is indeed indicative for competence development, but the strength of regulation by individual csRNAs is apparently not correlated with predicted complementarity.

At the moment, we cannot offer a final explanation for this discrepancy. Although the region of csRNA–*comC* interaction is roughly defined by the *comC8* mutations, base pairing may not be precisely predicted by the applied program (Busch et al., 2008). In addition, it has been demonstrated in other sRNA-mediated regulations that only a core within a larger region of complementarity is critical (Balbontin et al., 2010; Papenfort et al., 2012). Structural probing *in vitro* and more detailed mutagenesis *in vivo* will be required to define residues important for csRNA control of *comC*.

Another aspect of sRNA-mediated regulation is certainly the relative abundance of both partners. Since it is generally assumed that sRNAs are eliminated in the course of regulation (Gottesman and Storz, 2011; Storz et al., 2012), their expression level is of outstanding importance (Levine and Hwa, 2008). Based on previous northern blot analyses, a strong difference in expression was not detected for individual csRNAs (Halfmann et al., 2007b; Schnorpfeil et al., 2013), but subtle changes cannot be ruled out.

A further complication is also related to the consumption of sRNAs during regulation. Many sRNAs have been found to bind more than one target (Gottesman and Storz, 2011; Storz et al., 2012). All five csRNAs in *S*. *pneumoniae* regulate at least two more genes besides *comC* (Schnorpfeil et al., 2013) and it is most likely that there are further targets for the csRNAs. Depending on the expression level of these additional targets and the efficiency of csRNA binding to their mRNAs, single csRNAs may be titrated away from *comC* control.

While the reason of differential regulation of *comC* by single csRNAs remains elusive, it is clear that more than one csRNA is needed to prevent *S*. *pneumoniae* from reaching the competent state. Even two csRNAs were not enough, at least three csRNAs are needed. Of the three tested combinations, csRNA1,2,3 or csRNA1,2,4 were sufficient to block competence, but not the combination of csRNA1,2,5. It appears that each combination of three csRNAs among csRNA1,2,3, and 4 will be able to prevent natural transformation. Therefore, the role of each of these four csRNAs in competence regulation is clearly negative.

The role of csRNA5, however, appears to be more complex. The largest of the csRNAs acts positively on the level of early competence gene expression and only slightly suppressive on the timing of competence gene expression. This positive effect cannot be explained by regulation of *comC*, but is most likely due to the regulation of unidentified target(s) for csRNA5. Early competence gene expression is under feed-back control of a late competence gene product DprA (Mirouze et al., 2013; Weng et al., 2013). Inactivation of *dprA* results in a strong increase of early gene expression, but additionally late genes are also overexpressed. Quite in contrast, the observed effect of csRNA5 on competence gene expression is restricted to early competence genes (**Figures 1B–D**). Therefore, it appears unlikely that *dprA* is the unknown target for csRNA5. It has also been described that an intrinsic mechanism dependent on non-phosphorylated ComE could restrict especially transcription from the *comCDE* promoter (Martin et al., 2013). However, the effect of csRNA5 was virtually identical for both, the *comC* and the *comX* promoter (**Figures 1B,C**). It appears therefore, that csRNA5 regulates a novel process in early competence gene regulation.

The special role of csRNA5 in early competence gene expression adds an interesting twist to csRNA-mediated competence regulation. Due to the downregulation of CSP production, the csRNAs are involved in setting the threshold for competence induction. Once threshold is reached, csRNA5 may ensure a robust response to CSP by enhancing early gene expression.

## Conclusion

The csRNAs are not a simple device to turn down competence, but interfere with competence regulation in a complex manner.

## Conflict of Interest Statement

The authors declare that the research was conducted in the absence of any commercial or financial relationships that could be construed as a potential conflict of interest.
